# Effects of the Chinese Herb Medicine Formula “She-Xiang-Yu-Hong” Ointment on Wound Healing Promotion in Diabetic Mice

**DOI:** 10.1155/2022/1062261

**Published:** 2022-01-29

**Authors:** Qingjie Li, Xinjun Liu, Shihui Yang, Chunrun Li, Wei Jin, Weiwei Hou

**Affiliations:** ^1^Hospital of Chengdu University of Traditional Chinese Medicine, Chengdu, China; ^2^State Key Laboratory of Southwestern Chinese Medicine Resources, School of Pharmacy, Chengdu University of Traditional Chinese Medicine, Chengdu, China

## Abstract

Wound healing in diabetic patients is a difficult problem to be solved at present. In addition, patients with diabetes have an increased risk of postoperative wound complications. “She-Xiang-Yu-Hong” (SXYH) ointment is a type of traditional Chinese medicine (TCM) compound used to treat wounds. Over the past few years, SXYH has been applied in the Affiliated Hospital of Chengdu University of TCM (Chengdu, China) for the treatment of diabetic foot infections and bedsores, whereas there has been rare research on the effect of SXYH ointment on wound healing. In this study, SXYH ointment was first applied to streptozotocin (STZ)-triggered diabetic ICR mice (4–6 weeks, 20 ± 2 g) to observe the accelerated wound healing and the shortened wound healing period. As indicated by the histology and biochemistry analyses of skin biopsies, the wounds treated using SXYH ointment showed an increase in the granulation tissue. Moreover, SXYH also modulated the inflammation response by regulating affinity proinflammatory cytokines release (e.g., IL-6 and TNF-*α*). Furthermore, SXYH ointment obviously improved collagen fiber deposition and tissue on the wound surface. On the whole, this study indicated that SXYH ointment could accelerate wound healing, promote blood vessel formation, and suppress inflammations. Thus, the clinical potential of SXYH ointment was demonstrated in the treatment of diabetes and refractory wounds.

## 1. Introduction

Wound healing injury caused by diabetes is one of its complications, and the clinical treatment of wound healing injury is challenging [1]. Skin wounds with full thicknesses cause damage to considerable structures, cell layers, and lineages. If the protective barrier is broken, the wound heals immediately as it is in the epidermis and dermis [2]. Wound healing can be achieved through many processes that are mainly divided into four stages, i.e., instant responses; inflammation responses; proliferating process, migrating, and contracting responses [3]; and resolution phase. During wound healing, the wound site can show levels of inflammation cells, cytokines, hormones, and collagen in the blood. Aggregating inflammation cell refers to the subsequent phase for chronic wound healing, in which the mRNA expressing state pertaining to inflammation factors covering interleukin-6 (IL-6) and tumor necrosis factor-*α* (TNF-*α*) is noticeably increased [4]. It is noteworthy that angiogenesis mainly affects the success of wound healing. It has the germination of capillaries at the wound edge and then invades the injured site [5]. After a few days, a network of microvessels develops across the wound area, providing nutrients and oxygen to the growing tissue and helping form a temporary wound matrix. Collagen refers to a vital matrix (ECM) outside cells for wound healing, with the quantity and classifications affecting wound healing speed. Abnormal arrangement of collagen, dysfunction of angiogenesis, and chronic inflammation are often found in the wound caused by diabetes [6]. Thus, the wound is difficult to heal rapidly [7].

Wound care represents a significant biomedical burden. Management and treatment of chronic wounds including venous leg ulcer and pressure ulcers have been reported to cost 2% to 3% of the healthcare budgets in developed countries and affect millions of people annually [8]. These numbers are rapidly growing worldwide due to increasing aging populations and sharp and rise incidence of pathologies such as diabetes, obesity, and cardiovascular diseases.

At present, conventional treating approaches, covering chemistry-related drugs and surgery, face difficulty in achieving satisfactory results. For this reason, feasible drugs for diabetic patients' rapid wound healing should be developed. Traditional medicines have always achieved an application for medicine and have attracted wide attention because of their ability of expediting wound healing with no serious side effect [9, 10]. The mentioned herbs can refer to subsequent medicine to heal wounds [11]. Therefore, combined with the demand of the market of pharmaceuticals, natural wound healing agent used in traditional medicine has made great progress in the academic world and become the key to the pharmaceutical industry [12–15].

**Figure 1:**
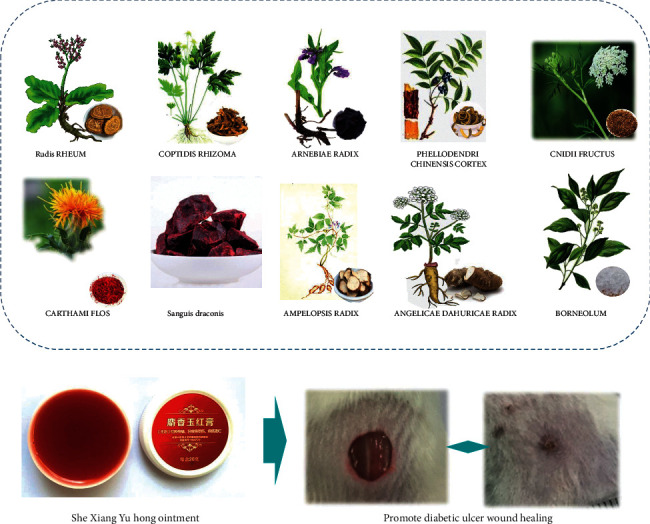
Schematic illustration of the topical therapy with “She-Xiang-Yu-Hong” ointment on wound healing promotion in diabetic mice.

SXYH is a kind of TCM compound for wound treatment. SXYH has been used in the treatment of diabetic foot infections and bedsores in the Affiliated Hospital of Chengdu University of TCM (Chengdu, China) for more than a decade (Figure 1), but there are limited studies on the effect of SXYH on wound healing. Therefore, the present study has been carried out to assess the efficacy of the SXYH ointment in a cutaneous punch wound model of streptozotocin-induced diabetic ICR mice. However, due to the complexity of the diabetic ulcer, a more macroscopic method was used for the comparative research, which included wound healing area, pathological sections, inflammatory factors, immunohistochemical method, and other methods, to assess the healing effect of SXYH ointment. Furthermore, the healing mechanism of the SXYH ointment was investigated preliminarily.

## 2. Materials and Methods

### 2.1. Chemicals and Biochemicals

Shanghai Yuanye Biotechnology Co., Ltd. (Shanghai, China) provided streptozotocin (STZ, batch number: S17049). The Affiliated Hospital of Chengdu University of TCM (Chengdu, China) offered SXYH ointment. Chengdu Kelong Chemical Co., Ltd. (Chengdu, China) provided other chemical reagents of analytical grade.

### 2.2. Animals

Male ICR mice (4–6 weeks) weighing 18–20 g originated from Si Pei Fu (Beijing, China) Experimental Animal Science and Technology Co., Ltd. The raising of all mice was achieved rigorously abiding by international ethical instructions and National Institutes of Health instructions on the care and use of laboratory animals. Mice were raised in (22–24°C) rooms at controlled temperatures. A 12-hour light/dark cycle was conducted, with food and water provided as needed. Forty mice were used in the experiment. All animal experiment procedures followed Chengdu University of Traditional Chinese Medicine Animal Welfare Guidelines (animal experiment ethics approval number: 2021DL-004).

### 2.3. *In Vivo* Wound-Healing Experiments

The model of diabetic ulcer in mice was established by referring to the relevant literature [11, 16]; the researchers induced a diabetic model by intraperitoneally injecting 240 mg/kg STZ. The blood glucose level was determined using a blood glucose meter (Sannuo, Anzhu, China). On the 3rd day after the injection, the blood glucose level of STZ-treated mice was higher than 400 mg/dL, and the STZ-treated mice were used as a diabetes model for the subsequent study. Mice received the anesthetization using isoflurane. The wound model was built. A 1 cm puncture was used to create a 1.0 cm round wound with full thickness in the muscular layer of the back. The animals were then randomly divided into two groups (*n* = 32) and assessed for 21 days in accordance with the standard protocol. The animals were treated with ointment preparation in the absence (control group) and SXYH ointment. The ointment base containing the same formulation as the study product without the active agent was used in the vehicle control group. For this reason, an ointment base with the identical formulation as the study product was applied in the experimental design, whereas no active agent was used.

### 2.4. Wound Healing Rate Determination

The researchers used SXYH for covering the wound region for 21 days, while the control groups and the vaseline groups were not treated. The dynamics of wound closure were determined by digital photography. The area of the wound and the rate of wound closure were measured immediately after the wound healed, and the area and rate of wound closure were measured daily for 21 days. The calculation method of wound closure rate is written as wound closure rate = (original wound area − open area on the test day)/original wound area ×100%.

### 2.5. Histological Analysis

One-third of the mice were euthanized and sacrificed in their respective groups on day 3, day 7, day 14, and day 21 after the injury. Wound lesion tissues were excised and fixed throughout the night in 4% buffered formalin solution and then embedded within paraffin. Tissue sections (5 *μ*m) were stained with hematoxylin and eosin (H&E) for the morphology evaluation. Using Masson's trichrome stain kit (Sigma), the researchers conducted an analysis on collagen of skin wounds.

### 2.6. Cytokine Measurements

The wound biopsies (*n* = 8) treated with the vehicle control of SXYH ointment received the homogenizing process, centrifugation at 1500 g, and storing process at −80°C till being assayed. The researchers employed the homogenate fluid generated for measuring TNF-*α* and IL-6 by the enzyme-linked immunosorbent assay (ELISA) technique with the use of specific antibody (purified and biotinylated) and cytokine standards, in accordance with the instructions of the producer. Optical densities were tested at 450 nm in a microplate reader. The cytokine level had the expression in pg, and sensitivities were >10 pg/mL.

### 2.7. Immunofluorescence Staining of Angiogenesis

Blocked paraffin sections (5 mm) received the baking, deparaffinizing, and rehydrating processes. Next, the tissues received the heating in a citrate buffer. It was cooled and then treated using 3% H_2_O_2_. Subsequently, it was incubated using 3% albumin from bovine serum (Servicebio, Wuhan, China). The samples received the incubation, respectively, using monoclonal primary anti-CD31 antibody (1 : 500; Servicebio, Wuhan, China) and polyclonal primary anti-VEGF antibody (1 : 4000; Abcam, Shanghai, China) throughout the night. Lastly, all slices received the staining process using 3, 3-diaminobenzidine tetrahydrochloride and substrate (Dako, Shanghai, China), washing, dehydrating, air-drying, and fixing using neutral resin. The stained section received the visualization based on a light microscope at magnification 400×. The researchers obtained the images with the use of the refrigeration CCD imaging system (OPTEC DV330).

### 2.8. Statistical Analysis

All data were mean ± SD. Two-way ANOVA was used for analyzing the results. Dunnett's test was used for accessing the effect of time and treatments. Differences were considered significant if *P* < 0.05.

## 3. Results and Discussion

### 3.1. Significant Reduction of Wound Size Treated Using SXYH Ointment

**Figure 2:**
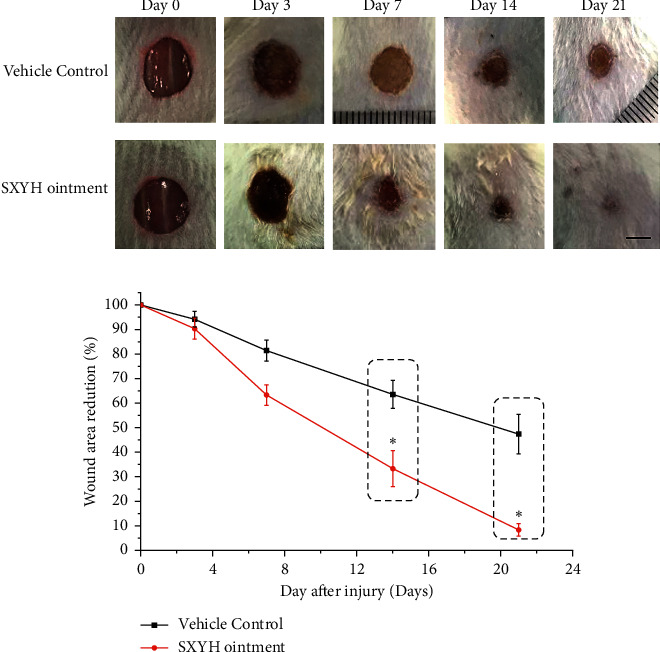
Topical application of SXYH ointment accelerates wound closure in full-thickness excisional wounds. (a) Representative photographs taken from full-thickness wounds of mice. (b) Rate of wound closure induced by topical application of ointment base (the vehicle control group) and SXYH ointment at days 3, 7, 14, and 21. Data are expressed as percentage of reduction area from the original wound size (day 0). Values are mean ± SD (*n* = 8 wounds/group) (^*∗*^*P* < 0.05).

For estimating the wound repair capability of treatment in diabetic mice, the researchers used the SXYH for wounds in mice. Next, the macroscopic research was carried out (Figure 2). In the excisional wounds, better skin appearance was noted in the SXYH ointment group. The wound healing rate of the mice received the assessment by the percentage of wound surface covered by regenerating epidermis. In Figure 2(b), the wound healing rate in the SXYH ointment group received the significant acceleration as compared with the vehicle control group at 7 days (^*∗*^*P* < 0.05).

SXYH ointment refers to a TCM effectively exploited to treat chronic ulcers in diabetic patients. However, the effect of SXYH ointment on diabetic wound surface has not been demonstrated. Diabetes causes many serious clinical complications, including chronic ulcers. Our study showed that SXYH ointment accelerated wound closure and shortened healing period in STZ-triggered diabetic mice. Thus, SXYH ointment is a potential strategy for the treatment of diabetic wounds.

### 3.2. Remarkable Improvement of Reepithelialization and Granulation Tissue Formation in the Wounds Treated Using SXYH Ointment

**Figure 3:**
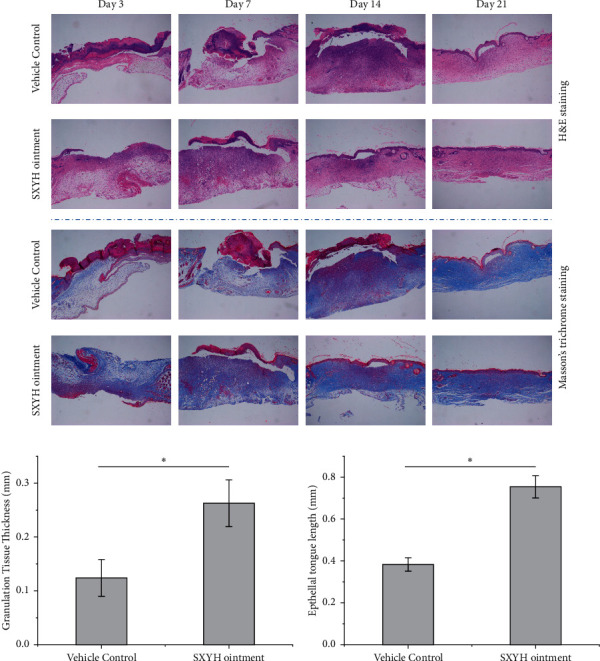
(a) The effect of the ointment on epithelium proliferation and collagen deposition in excision wounds with full thicknesses by H&E and Masson's trichrome staining (40×). (b) Quantitative analysis of granulation tissue thickness after 7 days' wounding. (c) Quantitative analysis of epithelial crawling distance after 7 days' wounding (^*∗*^*P* < 0.05).

To gain insights into the effect exerted by SXYH ointment treatment on wound healing, the epidermal migration and collagen deposition, which are the most common indications in wound healing, were observed. Masson's trichrome staining was performed to analyze the excision wounds with full thicknesses. As indicated from the results, the thickness of the epidermis in the SXYH ointment group noticeably grew as compared with that of the vehicle control group at day 7, and the length of the newly formed epithelium in the SXYH ointment-treated group was noticeably longer than that in the vehicle group at day 7, separately (Figure 3). Next, the collagen fibers in the SXYH ointment group were more extensive and orderly arranged than those in the untreated vehicle control group, showing more blue area at day 7 or day 14. Besides, the collagen deposition at wounds displayed the time-dependent characteristic, with the treated group at day 14 displaying more collagen levels.

### 3.3. Cytokine Release in the Skin Wound Biopsies

**Figure 4:**
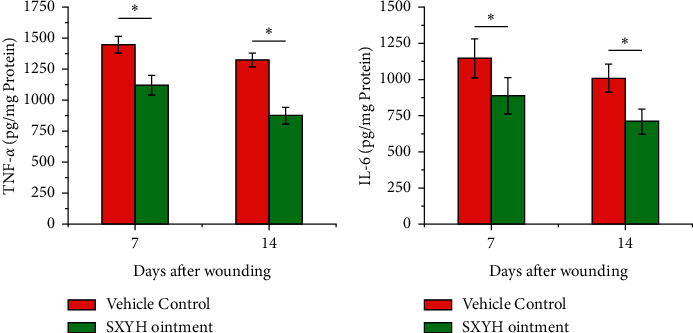
Effects of SXYH ointment in cytokines production in the skin wound biopsies Tissue homogenates were prepared from the wound biopsies obtained from animals at days 7 and 14 that were treated with SXYH ointment and with the vehicle control. (a) TNF-*α* and (b) IL-6 were assayed by ELISA. Data are mean ± SD (*n* = 8 wounds/group). ^*∗*^*P* < 0.05 compared to the vehicle control group.

After the wounding process, TNF-*α* and IL-6 in the wound tissue in the SXYH ointment group had downregulated expressions. The current levels of TNF-*α* and IL-6 were a key factor in the persistent inflammation response in the wound of diabetic patients [17]. TNF-*α*, a secreted protein, exhibits numerous cellular functions (e.g., the control of apoptosis, cell differentiation, and cell proliferation as well as cell growth) [18]. IL-6, a major constituent of the contractile apparatus, is extensively used as a marker of myofibroblast formation [19, 20]. It was broadly indicated that inhibition of TNF-*α* and IL-6 wound reduce inflammation and promote wound healing. In accordance with Figures 4(a) and 4(b), the concentration of TNF-*α* and IL-6 cytokines obtained within the tissue homogenates prepared from the wound biopsies obtained from animals at day 7 and day 14 treated using SXYH ointment showed a noticeable decrease of the abovementioned cytokines, compared to the vehicle control group (*P* < 0.05).

Inflammation is considered to be an important step in the wound healing because it prepares the environment for the wound healing [21]. However, an excessive inflammation response may delay wound healing. In the first stage of healing, regulation of cytokines release, including IL-6 and TNF-*α*, affects various processes at the wound site, including stimulation of keratinocyte and fibroblast proliferation, synthesis and breakdown of extracellular matrix proteins, fibroblast chemotaxis, and regulation of immune responses. The abovementioned results indicate that SXYH ointment may regulate inflammation response through the inhibition of the chemotaxis of inflammation cells and proinflammatory cytokines release including IL-6 and TNF-*α* from the wound surface. Thus, the extent and duration of the inflammation response can be controlled, which contributes to the successful closure of the wound.

### 3.4. SXYH Ointment Accelerated Angiogenesis

**Figure 5:**
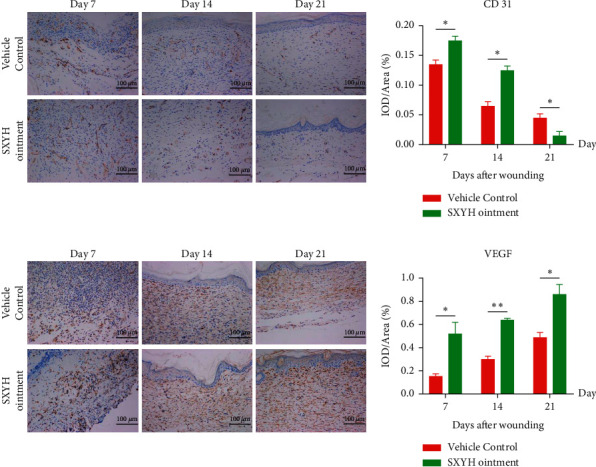
The SXYH ointment rapidly advances angiogenesis in the wounds as compared to the vehicle control. Sections of wounds harvested on days 7, 14, and 21 postwounding were immunostained with anti-CD31 (a) or with anti-VEGF (b).

Angiogenesis is of high significance to wound healing, which creates essential nutrients for skin tissue and supporting the formation of skin structure [22, 23]. Vascular endothelial growth factor (VEGF) refers to a vital regulatory factor of physiological and pathological angiogenesis, which can regulate the migrating process, proliferating process, and angiogenesis pertaining to vascular endothelial cells during wound healing [24]. Loss of VEGF may be one of the main reasons for reduced neovascularization and delayed wound healing in diabetic patients. Platelet colorectal cancer cell adhesion molecule-31 (CD31) is expressed in close connection of skin endothelial cells, which is involved in angiogenesis [25]. Therefore, CD31 can also indicate the level of angiogenesis. In order to confirm whether SXYH ointment can promote angiogenesis in diabetic patients, we used immunohistochemical staining to detect the expression of the abovementioned three proteins [18]. As shown in Figure 5, SXYH ointment increased the positive expression of CD31 and VEGF in newly healed skin tissue by 7 and 14 days compared with the drug-loaded control group. The number of new blood vessels is proportional to the number of CD31, so the number of CD31 is also decreasing at later stage. The expression of VEGF increased with the increase of the number of vessels, so the content of VEGF increased gradually.

The formation of new blood vessels through angiogenesis is the key to wound healing. Insufficient angiogenesis not only leads to reduced blood flow and oxygen but also restricts the entry of inflammation cells including macrophages into the wound, leading to delayed wound healing [26]. In the wound treated using SXYH ointment, the formation of new blood vessels was more obvious than in the control group. This indicates that angiogenesis improved by SXYH ointment rapidly responds to the metabolic demands of new tissue, ultimately leading to accelerated wound healing.

Treating wounds caused by diabetes can be difficult. The results of this study indicate that SXYH ointment can inhibit inflammation, improve angiogenesis, and regulate collagen. Thus, treatment with SXYH ointment proved to be a promising method for STZ-triggered wound healing in diabetic mice. In diabetics, delayed wound healing leads to chronic nonhealing ulcers that may require amputation, which can be expensive. Therefore, the use of effective dressings for the mentioned wounds can provide effective health benefits. The data presented here indicate that SXYH ointment can indeed meet this urgent need for clinical application.

SXYH ointment has been applied for treating clinical diabetic ulcers in the Affiliated Hospital of Chengdu University of Traditional Chinese Medicine as a preparation of Chinese medicine hospitals. However, the use of the ointment has some drawbacks. If it is used improperly, it may cause bacterial infection. Thus, some new topical preparations (e.g., liposomes, hydrogels [27–29], and nanofibers [30, 31]) can be studied. The development of new preparations helps solve the problems of administration difficulties of topical ointments applied for exposed wounds, which may easily cause secondary infections. In addition, the quality control of SXYH ointment still stays at the content determination of a single component. At the later stage, the content of the effective components of multiple medicinal materials should be tested. If necessary, a fingerprint is required to control the quality of SXYH ointment. Lastly, the mechanism of SXYH ointment in treating diabetic ulcers remains unclear. The subsequent research will use transcriptomics or proteomics to further study the mechanism of SXYH ointment in treating diabetic ulcers.

## 4. Conclusion

It was first proved that SXYH ointment had a positive effect on the wound healing of type I diabetic mice. SXYH can accelerate wound healing, promote vascularization, and inhibit inflammation. The wound healing effect of SXYH ointment may be the result of VEGF alone or in addition. The exact mechanism by which SXYH ointment acts on endothelial cells, fibroblasts, and keratinocytes remains to be studied. Thus, SXYH ointment has proved to be a promising treating approach for topical application in the treatment of skin tissue injuries, primarily chronic wounds.

## Data Availability

The data used to support the findings of this study are available from the corresponding author upon request.
